# Dynamic coagulofibrinolytic responses under long-term VV-ECMO management without anticoagulation in a COVID-19-ARDS patient: A case report

**DOI:** 10.1097/MD.0000000000032817

**Published:** 2023-01-27

**Authors:** Hironori Matsumoto, Satoshi Kikuchi, Satoru Murata, Muneaki Ohshita, Yutaka Harima, Suguru Annen, Naoki Mukai, Yuki Nakabayashi, Shirou Ogawa, Mitsuo Okita, Jun Takeba, Norio Sato

**Affiliations:** a Ehime University, Graduate School of Medicine, Department of Emergency and Critical Care Medicine, Toon City, Ehime, Japan.

**Keywords:** fibrin/fibrinogen degradation products (FDP), plasmin-α_2_-plasmin inhibitor complex (PIC), soluble fibrin (SF), thrombin-antithrombin complex (TAT), thromboelastography

## Abstract

Venovenous extracorporeal membrane oxygenation (ECMO) is recommended for the treatment of critically ill patients with acute respiratory distress syndrome due to coronavirus disease 2019 (COVID-19). However, ECMO management can cause both bleeding and thrombotic complications. There are insufficient coagulofibrinolytic data for appropriate ECMO management in patients with COVID-19.

**Patient concerns:**

A 48-year-old man with severe COVID-19-acute respiratory distress syndrome underwent long-term venovenous ECMO management for 48 days. Refractory oronasal bleeding developed on day 13, so the administration of unfractionated heparin was ceased for 29 days.

**Diagnosis:**

The patient showed dynamic coagulofibrinolytic responses associated with ECMO management, as shown by fibrin/fibrinogen degradation products, soluble fibrin, thrombin-antithrombin complex, and plasmin-α_2_-plasmin inhibitor complex elevations, suggesting the development of ECMO-induced coagulopathy.

**Interventions:**

We assessed coagulofibrinolytic markers to decide the appropriate timing for controlling excessive activation of coagulation by exchanging ECMO circuits. Moreover, viscoelastic hemostatic assays were used for adequate transfusion of blood products.

**Outcomes:**

Safe long-term ECMO management was completed, which was withdrawn on day 48. The patient was weaned off mechanical ventilation on day 57 and was transferred to another hospital for rehabilitation.

**Lessons:**

Monitoring the coagulofibrinolytic status using markers and viscoelastic hemostatic assays may be effective for safe long-term ECMO management even without anticoagulant therapy.

## 1. Introduction

Venovenous extracorporeal membrane oxygenation (VV-ECMO) is recommended for the treatment of severe and critically ill patients with acute respiratory distress syndrome (ARDS) due to coronavirus disease 2019 (COVID-19).^[1]^ Generally, ECMO management for patients with severe ARDS includes continuous and systemic anticoagulation to prevent thrombosis of the ECMO circuit.^[2]^ However, ECMO management causes both bleeding and thrombosis, and further COVID-19-induced coagulopathy appears to complicate both manifestations. There is insufficient clinical data that include coagulofibrinolytic responses to establish management for hemostasis including anticoagulation in patients with COVID-19 receiving ECMO.^[1]^ Herein, we report the case of a COVID-19-ARDS patient who received long-term VV-ECMO management without anticoagulant therapy because of a refractory bleeding complication. Our case illustrates the dynamic coagulofibrinolytic response that might indicate the transition from hemostasis to systemic coagulopathy. The clinical findings of our case were consistent with the definition of disseminated intravascular coagulation (DIC).^[3]^ Assessment of the coagulofibrinolytic condition using markers and TEG6s^®^ was effective in minimizing thrombotic and bleeding complications.

## 2. Case presentation

A 48-year-old man with a height of 171 cm and a body weight of 87 kg presented with fever (38°C). Four days after symptom onset, the patient was admitted to an emergency hospital with progressive dyspnea and diagnosed with COVID-19 pneumonia based on severe acute respiratory syndrome coronavirus 2 polymerase chain reaction. Chest computed tomography (CT) scan revealed bilateral ground-glass opacities and consolidation. His pneumonia worsened under oxygen therapy and the administration of steroids and remdesivir for 8 days; therefore, he was intubated and transferred to our university hospital, the local designated center for patients with COVID-19-ARDS in Ehime, Japan.

**Figure 1.**
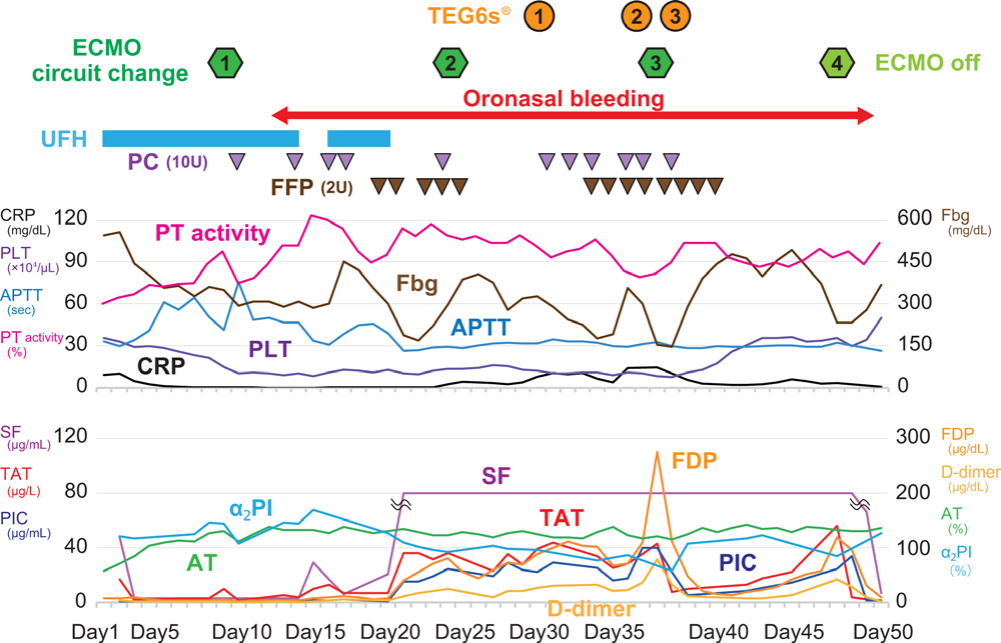
Time course of coagulofibrinolytic parameters and ECMO management. ECMO = extracorporeal membrane oxygenation.

On arrival (considered as day 1 for the case timeline, the time course related to the diagnoses and interventions is depicted in Fig. 1), his respiratory condition further deteriorated and he was not able to maintain a sufficient oxygenation level despite high driving pressure and end-expiratory pressure ventilation (PaO_2_/FiO_2_ ratio 61.9, PaCO_2_ 51.9 mm Hg). Thus we decided to establish VV-ECMO. Cannulation was carried out using a 25 Fr drainage cannula via the right femoral vein and a 19 Fr inflow cannula via the right jugular vein. Initial ECMO settings were sweep gas flow 5.0 L/min, FiO_2_ 100%, blood flow 5.3 L/min, and rotation speed 2050 rpm, and regulated according to his respiratory status. We commenced continuous administration of unfractionated heparin (UFH) titrating to achieve an activated partial thromboplastin clotting time (APTT) ranging from 50 to 60 seconds, although anti-factor Xa monitoring was not available in our hospital. While on ECMO we performed very low-pressure and low-volume ventilation to provide “lung rest,” and also provided prone positioning.

After the initiation of ECMO management, it took time to achieve the target control of APTT. On day 9, hemolysis occurred with minimal coagulofibrinolytic activation (fibrin/fibrinogen degradation products [FDP] 5.8 µg/mL, soluble fibrin [SF] < 3 µg/mL, thrombin-antithrombin complex [TAT] 10.3 µg/L, plasmin-α_2_-plasmin inhibitor complex [PIC] 1.6 µg/mL). An ECMO circuit change resolved hemolysis, and apparent thrombus formation was observed within the pump head. Subcutaneous emphysema developed after the circuit change. Contrast-enhanced CT revealed subcutaneous and mediastinal emphysema, but no thrombus formation. After tracheostomy on day 13, refractory oronasal bleeding was caused by contact with the feeding tube and the tracheal tube developed (APTT 51.9 seconds, fasting blood glucose: fibrinogen 310 mg/dL, platelet [PLT] count 10.3 × 10^4^/µL). Although the patient had had negative severe acute respiratory syndrome coronavirus 2 polymerase chain reaction results twice, the bleeding persisted despite cauterization and oronasal packing; therefore we decided to discontinue the administration of UFH on day 20. After that, the coagulofibrinolytic markers increased and sustained elevated levels (APTT 25.8 seconds, FDP 41.8 µg/mL, SF > 80 µg/mL, TAT 36.6 µg/L, PIC 15.9 µg/mL on day 21). On day 24, we performed a planned ECMO circuit change, and minimal areas of peripheral thrombus formation were observed on the oxygenator membrane. Although the increased coagulofibrinolytic activity mildly improved, oronasal bleeding continued despite treatment and transfusion of plasma and PLTs for hemostasis. We evaluated the coagulofibrinolytic status using TEG6s^®^. It revealed mild hypocoagulability even under transfusion, and fibrinolytic shutdown whereas the markers showed increased fibrinolytic activity (Table 1). These results indicated that the bleeding tendency was caused by hypocoagulability without hyperfibrinolysis; therefore we planned to transfuse more aggressively.

**Table 1:** TEG6s^®^ and coagulofibrinolytic biomarkers on day 30, 37, and 38.

		Normal range	❶ Day 30	❷ Day 37	❸ Day 38
Biomarkers	PLT × 10^4^/μL	15.0–40.0	12.5	8.4	11.1
	Fbg mg/dL	200–400	328	155	291
	PT activity %	80.0–120.0	101.1	81.9	103.3
	TAT μg/L	<3.0	39.3	43.5	10.3
	SF μg/mL	<7.0	>80	>80	>80
	PIC μg/mL	0.0–0.8	22.4	>40	5.8
	FDP μg/mL	<5.0	89.5	275.0	48.6
	D-dimer μg/mL	<1.0	27.7	79.0	12.6
TEG6s	R (CKH) min	4.3–8.3	4.8	7.3	4.6
	R (CRT) min	0.3–1.1	0.5	2.8	0.8
	K (CKH) min	0.8–1.9	2.5	5.8	2.3
	K (CRT) min	0.8–2.7	3.1	5.8	3.0
	A (CKH) deg	64–77	67.1	49.0	67.9
	A (CRT) deg	60–78	68.9	43.8	67.0
	MA (CKH) mm	52–69	51.5	35.4	49.3
	MA (CRT) mm	52–70	51.0	35.0	48.9
	MA (CFF) mm	13–30	11.4	2.1	13.5
	LY30 (CK) %	0.0–2.6	0.0	0.0	0.1

A= alpha angle, CFF= citrated functional fibrinogen, CK= citrated kaolin, CKH= citrated kaolin heparinase, CRT= citrated rapid TEG, Fbg = fasting blood glucose, FDP = fibrin/fibrinogen degradation products, K=kinetics, LY30=Lysis at 30 minutes, MA= maximum amplitude, PIC = plasmin-α_2_-plasmin inhibitor complex, PLT = platelet, PT = prothrombin time, R=reaction time, SF = soluble fibrin, TAT = thrombin-antithrombin complex.

On day 37, an abrupt further FDP elevation with coagulofibrinolytic activation (FDP 275.0 µg/mL, SF > 80 µg/mL, TAT 43.5 µg/L, PIC 40.0 µg/mL) and consumption of coagulation factor and PLTs (fasting blood glucose 155 mg/dL, PLT 8.4 × 10^4^/µL) were observed. Considering that it was necessary to correct the increased coagulation activity without resuming UFH for refractory bleeding, we changed the ECMO circuit. The coagulofibrinolytic activation subsequently improved and the FDP value decreased to a normal level. Only minimal areas of peripheral thrombus formation were observed on the oxygenator membrane. The evaluation using TEG6s^®^ before and after the ECMO circuit change confirmed the improvement in coagulability with the transfusion of plasma and PLTs against consumption coagulopathy (Table 1).

After the third circuit change, a gradual increase in the FDP level was observed again (FDP 119.8 µg/mL, SF > 80 µg/mL, TAT 56.1 µg/L, PIC 25.0 µg/mL). Improvement in the increased coagulation activity was thought to be required; however, ECMO was withdrawn due to improvement in the respiratory condition, and the patient was successfully decannulated on day 48. Apparent thrombus formation was observed within the pump head and the oxygenator membrane. Coagulofibrinolytic activation was normalized immediately after withdrawal from ECMO, and the oronasal bleeding also improved.

The patient was weaned off mechanical ventilation on day 57 and transferred to another hospital for rehabilitation. No obvious thrombotic findings were detected on CT images during the course of the treatment.

## 3. Discussion

In this study, we found that monitoring coagulofibrinolytic status using markers and viscoelastic hemostatic assays (VHAs) may have been effective for long-term safe ECMO management in a patient with severe COVID-19 ARDS who could not use anticoagulation due to oronasal bleeding. ECMO has been considered a candidate for the effective management of patients who do not respond to optimal conventional mechanical ventilation in COVID-19.^[1]^ In such situations, thrombosis is one of the main complications during ECMO management; therefore continuous anticoagulation is recommended.^[2]^ Paradoxically, a high risk of bleeding complications coexists; therefore, adequate anticoagulation management is required. In the previous reports, the median duration of ECMO for COVID-19 was reported to be 13.9 days.^[4]^ Some reports have demonstrated that good clinical outcomes can be achieved even after long-term ECMO treatment.^[5]^ Furthermore, a systematic review of anticoagulant-free ECMO revealed an incidence of circuity and patient thrombosis comparable to those in patients receiving continuous systemic anticoagulation.^[6]^ However, the median duration of ECMO management without any systemic anticoagulation was 4.75 days in this review, which was much shorter than the 29 days in our case.

The patient’s hemostatic condition under anticoagulant therapy was controlled within the localized thrombus formation without a systemic coagulofibrinolytic response. After the discontinuation of anticoagulation, systemic coagulofibrinolytic activation coexisting with bleeding and thrombotic complications occurred, which was in accordance with the definition of DIC, namely, “an acquired syndrome characterized by the intravascular activation of coagulation with loss of localization arising from different causes,” as proposed by the International Society on Thrombosis and Haemostasis.^[3]^ Under this situation, abrupt FDP elevations with marked coagulofibrinolytic activations were detected, which might indicate the transition from hemostasis to systemic coagulopathy. When blood is exposed to the non-endothelial surface of the ECMO circuit, hemostatic alterations including coagulation activation and inflammation occur. Hemostatic equilibrium under ECMO is maintained by reserve capacity and administration of an anticoagulant agent. However, the synergistic effect of endothelial dysfunction and inflammation due to causative or concomitant diseases interferes with the balance and causes disequilibrium with resultant bleeding or thrombosis: ECMO-induced coagulopathy (EIC).^[7]^ Furthermore, extensive cross-talk between coagulation and inflammation caused by continuous exposure to ECMO circuit and causative disease can amplify each other and evoke exacervation of EIC.^[8]^ Particularly in long-term ECMO management, EIC may eventually be complicated by dysregulation of coagulation activation, malfunction of fibrinolysis, consumption coagulopathy, and impairment of anticoagulant systems, leading to a final common pathway of fulminant coagulation failure and the clinical presentation of DIC. The pathophysiology of EIC is likely to be heterogeneous and multifactorial. Acquired von Willebrand syndrome caused by the loss of high-molecular-weight multimers of von Willebrand factor due to high shear stress under ECMO, complications associated with heparin-induced thrombocytopenia and thrombotic microangiopathy, and many other factors can be potential mechanisms of thrombotic and bleeding events during ECMO management.^[9,10]^ In the current case, COVID-19-associated coagulopathy, inflammation, and endothelial cell dysfunction may have affected the EIC. ECMO circuit changes and withdrawal drastically improved the systemic coagulofibrinolytic activation and refractory oronasal bleeding. These responses demonstrate that prolonged ECMO management without anticoagulation is significantly associated with excessive coagulofibrinolytic activation, and the markers sensitively represented the coagulofibrinolytic fluctuation.

VHAs are whole-blood point-of-care coagulation assays used to measure the viscoelastic properties of clots. Currently, the use of VHAs is recommended to guide the administration of blood products and coagulation factors in the presence of bleeding. Recent studies have suggested that hypercoagulable states, as demonstrated by VHAs, can predict the risk of thrombotic complications.^[11]^ In the current case, TEG6s^®^ was effective for adequate transfusion of plasma and PLTs from a functional aspect. It should be noted that there are some differences between the VHAs and coagulofibrinolytic markers. VHAs indicate hemostatic functional ability, whereas markers such as TAT, SF, PIC, and FDP represent coagulation and fibrinolysis activity. As described above, we should detect and improve excessive coagulation activation, and in this regard, markers are likely to be able to detect this response more sensitively than VHAs. Actually, in a previous report of cardiovascular surgery, coagulofibrinolytic markers including TAT reflected surgical stress and were associated with bleeding volume.^[12]^ Although UFH could not be started because of the patient’s bleeding tendency, long-term ECMO management was made possible in this case by controlling the excessive activation of coagulation by exchanging circuits at the required time. Considering the social aspects of the COVID-19 epidemic and treatment cost, optimal anticoagulant management for COVID-19-associated coagulopathy, and EIC should be established. Under such circumstances, monitoring VHAs for adequate transfusion of blood products and markers for controlling coagulofibrinolytic activation may be an optional method for the safe and adequate management of ECMO.

## 4. Conclusion

The patient with severe COVID-19 ARDS presented herein exhibited unique coagulofibrinolytic responses under long-term VV-ECMO management without anticoagulation: abrupt coagulofibrinolytic activation with bleeding and thrombotic complications developed from hemostasis to systemic coagulopathy. Monitoring coagulofibrinolytic status using markers and VHAs may be effective for safe ECMO management in such situations.

## Acknowledgments

The authors would like to thank the nursing staff of the Intensive Care Unit 2 at Ehime University Hospital for their assistance.

## Author contributions

**Conceptualization:** Hironori Matsumoto, Satoshi Kikuchi, Satoru Murata, Muneaki Ohshita, Yutaka Harima, Suguru Annen, Naoki Mukai, Yuki Nakabayashi, Shirou Ogawa, Mitsuo Okita.

**Supervision:** Norio Sato.

**Writing – original draft:** Hironori Matsumoto.

**Writing – review & editing:** Jun Takeba.
